# The Healthy Cooking Index does not Predict the Carotenoid Content of Home-Cooked Meals

**DOI:** 10.3390/nu12020524

**Published:** 2020-02-19

**Authors:** Margaret Raber, Karen Basen-Engquist, Nancy E. Moran, Joya Chandra

**Affiliations:** 1 University of Texas MD Anderson Cancer Center, Houston, TX 77030, USAkbasenen@mdanderson.org (K.B.-E.); 2 Department of Pediatrics, Baylor College of Medicine, USDA/ARS Children’s Nutrition Research Center, Houston, TX 77030, USA; nancy.moran@bcm.edu

**Keywords:** food preparation, carotenoids, nutrition education, cancer survivorship

## Abstract

Home cooking programs are an increasingly popular approach to nutrition education and have the potential to promote diet quality among pediatric cancer survivors. A cornerstone of many programs is the use of fresh fruits and vegetables, which may support increased intake of many food components, including carotenoids, to improve survivor health. However, most dietary carotenoids in the United States currently come from processed vegetables, and it is unclear if the emphasis on fresh fruits and vegetables common in cooking education programs is associated with the total carotenoid content of meals. The objective of this analysis is to examine the relationship between fresh produce usage, practices commonly taught in healthy home cooking classes, and the carotenoid content of prepared meals among 40 parents with school-aged children. This is a secondary analysis of an observational study examining the quality of home cooking practices using an evidence-based index of behaviors, the Healthy Cooking Index (HCI). Nutrition-optimizing cooking practices, as quantified by the HCI, were not associated with the carotenoid content of meals (r = −0.24, *p* = 0.13). Further, total fruit and vegetable content of meals was not associated with total carotenoids (r = 0.14; *p* = 0.38), indicating heterogeneity in the carotenoid profiles of foods used by this population. High-carotenoid meals tended to use more canned and/or frozen tomato and vegetable products, and carotenoid content was associated with meals with sugar (r = 0.32; *p* = 0.04), and servings of refined grains (r = 0.49; *p* < 0.01). Our findings indicate an opportunity to educate pediatric cancer survivors and families on the incorporation of high-carotenoid food products while reducing refined grain and sweetener intake through a tailored home cooking intervention.

## 1. Introduction

Dietary patterns are increasingly appreciated to affect cancer prevention and survivorship [1]. Survivors of pediatric cancer are a particularly important target population for nutrition interventions, given the high survivorship rate, potentially long survivorship period, and iatrogenic effects of treatment. Survivors are at an increased risk, due to treatment and lifestyle factors, for several conditions that may be exacerbated by energy imbalance including cardiovascular disease, obesity, and secondary cancers [2,3]. Despite these health risks, pediatric cancer survivors have similar dietary habits to the general population, consuming too few fruits and vegetables, fiber, and whole grains, and excessive meat and sodium [4,5,6,7,8]. Thus, public health nutrition initiatives targeting the general population may offer amplified impacts in pediatric cancer survivor populations.

Food preparation (i.e., home cooking) education is an increasingly popular strategy used to promote good population nutrition, with programs being conducted in diverse locations across the United States for different target populations [9]. Programs specific to pediatric cancer patients and survivors have also been developed in an effort to help communicate nutrition recommendations for survivors and caregivers through practical skill-building [10,11]. To establish a quality metric for the home cooking practice outcomes of these rapidly expanding programs, the Healthy Cooking Index (HCI) was developed from a review of the existing literature on food preparation practices and health outcomes. The HCI was built to assess the presence or absence of cooking practices that are associated with higher diet quality (e.g., lower energy density and higher nutrient density) and reduced exposure to carcinogenic compounds [12]. Practices on the HCI include cooking “from scratch” (using basic ingredients rather than pre-made ingredients), using fresh produce, avoiding red and/or processed (cured, salted, etc.) meat, and incorporating whole grains [12]. Contemporary cooking programs focus on the use of fresh or frozen fruits and vegetables as a means of reducing added sugars and sodium that may accompany canned products. However, this approach may come at a cost to total intake of fruits and vegetables and intake of phytochemicals such as carotenoids by discouraging the use of potentially more convenient, canned products. Canned tomato and canned or frozen dark green leafy vegetable products are particularly concentrated sources of carotenoids due to the volume reduction that occurs with heat processing these foods.

Carotenoids, the red, orange, and yellow pigments abundantly found in fruits and vegetables are associated with a number of beneficial health outcomes for the general population, particularly with regard to cardiovascular and eye health (reviewed in [13]); growing evidence suggests particular benefits of consuming carotenoid-rich foods for cancer survivors [14,15,16,17]. In response to the evidence linking dietary patterns with higher intakes of fruits and vegetables and positive health outcomes, the 2015–2020 United States Department of Agriculture (USDA) Dietary Guidelines for Americans highlighted the importance of eating dark green, red, and orange vegetables, all of which are considered carotenoid-rich [18]. In addition to conveying biological effects, blood carotenoids serve as biomarkers of fruit and vegetable intake and dermal carotenoids are being actively investigated as a non-invasively monitored biomarker of fruit and vegetable intake [19,20,21,22,23]. Americans consume a variety of carotenoids, with the most abundant carotenoid in the diet being lycopene (5.5 +/− 0.2 mg/day), the red pigment found in tomatoes and watermelon, followed by beta-carotene (2.0 +/− 0.07 mg/day) [24]. Because canned tomatoes are a more concentrated form of tomatoes, they are the second most abundant vegetable in the American diet (after potatoes), and thermal processing, such as canning, increases lycopene bioavailability compared to fresh tomato products [25]. As such, processed tomatoes likely represent the major source of dietary carotenoid exposure for Americans [26,27].

Fresh ingredients, scratch cooking, and limiting processed foods are common themes in many cooking class interventions designed to improve diet quality and are captured by the HCI [10,12]. The HCI definition of processed foods is informed by the NOVA classification system which groups processed foods into three groups [12,28]. Groups 1 and 2 include unprocessed or minimally processed foods (such as milk, frozen vegetables, and hulled nuts) and processed culinary ingredients (such as flour, oil, and butter). Groups 3 and 4 include processed foods in which salt, sugar, or oil are added (such as canned or jarred vegetables and salted nuts) and ultra-processed foods (such as instant noodle packets). The HCI considers any ingredient in NOVA group 3 or 4 to be “processed food” and subsequent usage of the term “processed” in this communication is based on this definition.

However, some processed fruit and vegetables, in particular tomato products, are high in carotenoids, are widely used in the population, and offer a convenient and economical means of incorporating fruits and vegetables into the diet. Given the reality of high carotenoid concentration in some canned vegetable foods, an examination of the relationship between healthy cooking principles, total fruit and vegetable usage, and carotenoid content of home prepared meals will support the development of optimal strategies for cooking education among cancer survivor populations. Therefore, we sought to investigate (1) whether implementation of high HCI cooking practices are associated with carotenoid content of meals, and (2) the relationship between total carotenoid content and total fruit and vegetable content of prepared meals in a sample of parents with school aged children.

## 2. Materials and Methods

This report offers a secondary analysis of a previously completed observational study conducted from October 2017–June 2018. The details of that study have been detailed elsewhere [29], but are briefly reviewed below.

### 2.1. Participants

A convenience sample of 40 parent–child dyads were recruited in Houston and Austin, Texas. Approximately 25% of the sample included parents with pediatric cancer survivors at least one year off all treatment. Eligibility criteria included children being school-aged (5–17), parental ability to read and speak English, parent having reported cooking for the child at least one time per week on average, and no severe food allergies in the home. This study was reviewed and approved by the Institutional Review Board of the University of Texas MD Anderson Cancer Center (PA16-0995).

### 2.2. Data Collection

Each participant scheduled and completed a single home-cooking data collection session in their homes, during which one or two observers documented their actions while cooking as well as the ingredients and amounts used during food preparation. Home cooking sessions were video- and audio-recorded for analysis purposes. Participants were instructed to cook a typical meal, and describe their actions as they cooked. Observers were Master’s-level trained research staff with expertise in nutrition and behavioral science.

### 2.3. Analysis

The primary study analysis focused on exploring the relationship between observed overall cooking practices, produce usage, and the carotenoid content of meals. Exploratory analysis examined carotenoid content and ingredient usage by type of meal prepared. The resulting video and audio tapes of the session were examined along with observer notes and coded using the HCI: an evidence-based measure of healthy cooking practices commonly taught in formal healthy cooking programs [12]. Healthy cooking practices were defined as those that reduced energy density, increased nutrient density, and reduced potential carcinogen exposure. Less healthy cooking practices were defined as those that increased saturated fat, sugar, sodium, and potential carcinogen content of prepared meals. The HCI scoring system assigns +1 to healthy cooking practices and −1 to less healthy cooking practices. Points are then summed for an overall cooking quality score. Healthy cooking practices include cooking from scratch, using whole grains, citrus, herbs/spices, alliums, olive or canola oil, adding fruit and vegetables, measuring salt and fat, and using low fat cooking methods such as steaming and baking. Less healthy cooking practices include using red meat cooked at high temperatures to well done, adding sweeteners, using processed foods, animal fats, or processed meats, serving vegetables with creamy sauces, and using a deep frying cooking method.

Overall meal content was examined qualitatively and grouped into major categories based on the main dish prepared (e.g., pasta, chicken, tacos, etc.). Ingredients and amounts used by participants during food preparation were examined and analyzed for nutrient composition using the Nutrient Data System for Research software (NDSR 2017, University of Minnesota, Minneapolis, MN, USA). This process calculates full macro- and micronutrient profiles for individual servings of meals prepared by participants including individual and total carotenoid content. Other nutrition variables examined were total fruit and vegetables, carotenoid-rich vegetables (tomato, deep yellow vegetables, and dark green vegetables), fat, saturated fat, protein, sugar, fiber, energy density, and relevant vitamins and minerals. Correlations between carotenoid content of meals, cooking practices, and other nutrition variables were assessed using Pearson’s correlation coefficient and significance level set at *p* < 0.05. Demographic characteristics were examined using descriptive statistics. Comparative and descriptive statistics were all performed with SPSS (IBM SPSS Statistics for Windows, Version 25.0. Armonk, NY, USA: IBM Corp).

## 3. Results

Participant characteristics are shown in Table 1. Within the sample, the mean servings of combined fruit and vegetables in prepared meals was 2.74 (range = 0–9.46). Slightly less than half (46%) of vegetable servings were classified as “carotenoid-rich”, defined as being dark green, deep yellow, or tomato. Total carotenoid content per serving per meal varied widely in the population (range = 9 to 33,624 mcg), with lycopene content having the largest range (0 to 17,156 mcg).

Individual carotenoids were examined relative to carotenoid-rich vegetable content of meals. Individual carotenoids correlated, as expected, with specific categories of carotenoid-rich vegetables (Table 2) (i.e., lycopene and tomatoes r = 0.87; *p* < 0.01).

Total meal carotenoid content (sum of beta-carotene, alpha-carotene, beta-cryptoxanthin, lutein + zeaxathin, and lycopene) was positively associated with total servings of carotenoid-rich vegetables (dark green vegetables, tomatoes, and deep yellow vegetables) (r = 0.55; *p* < 0.01), but not with total fruit (r = −0.07; *p* = 0.67), total vegetables (r = 0.16; *p* = 0.32), or combined fruit and vegetables (r = 0.14; *p* = 0.38) (Table 3). Further, total carotenoid content was not associated with summative HCI score. These findings may be due to inter-individual heterogeneity in the carotenoid density of fruits and vegetables being used in meals, or carotenoid sources that may not be classified as fruit and vegetables (e.g., mixed dishes, shellfish, dairy).

**Figure 1:**
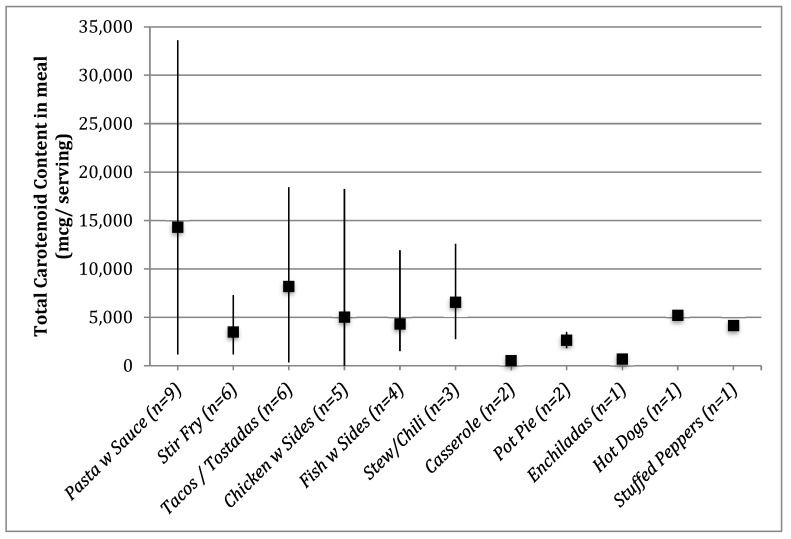
Scheme depicting means (as indicated by solid squares) and range (as indicated by solid lines) of carotenoid content per serving in prepared meals. () indicate the number of families that prepared a given category of dish.

Processed tomato products and other processed foods often contain added sugars or salt, and may not offer the same benefits of fresh fruit and vegetables such as fiber, vitamins and minerals. In order to better explore the sources of carotenoids in participant meals, prepared dishes were categorized by type. Although participants were instructed to make any meal of their choosing, all prepared meals could be grouped into one of 11 post hoc assigned meal types, with several participants opting to make pasta, tacos, or vegetable and meat stir-fry types of meals. This allowed for an examination of carotenoid variability within single meal types (Figure 1). The meals with highest and lowest carotenoid content in each meal type category were compared qualitatively (Table 4) in order to better understand potential strategies to increase carotenoid content in a given meal.

In order to determine if carotenoid content of meals was associated with other energy density, nutrients, and dietary variables, the associations of meal macro- and micronutrients with total carotenoid content, total fruit and vegetable usage, and carotenoid-rich fruit and vegetable usage were assessed (Table 5). This analysis revealed high carotenoid levels in meals were positively associated with sugar (r = 0.32; *p* = 0.04), and servings of refined grains (r = 0.49; *p* < 0.01) and sweets (r = 0.45; *p* < 0.01). Carotenoid totals were also positively associated with vegetable protein (*r* = 0.48; *p* < 0.01), vitamin A (retinol activity equivalents) (r = 0.54; *p* < 0.01), vitamin K (r = 0.051; *p* < 0.01), folate (*r* = 0.72; *p* < 0.01), iron (r = 0.66; *p* < 0.01), and selenium (r = 0.37; *p* = 0.02). This is unsurprising given that many carotenoid-rich fruit and vegetables contain these compounds.

## 4. Discussion

The carotenoid content of meals was not associated with HCI scores or total fruit and vegetable usage in this population. Through a more in-depth examination of prepared meals, high-carotenoid dishes appear to be driven by the incorporation of canned tomato products. Prepared meals that used canned or jarred vegetable products appeared to have higher carotenoid concentrations than those using only fresh vegetables. This was particularly true when meals contained canned tomato products. Although total carotenoid content was positively associated with some vitamins and minerals that are important to human health, they were also associated with sugar, refined grains, and sweets. Thus, cooking programs have an opportunity to educate families on the incorporation of carotenoid-rich processed foods while limiting added sugars and refined grains.

Not all fruits and vegetables contain high concentrations of carotenoids, and carotenoids may also be found in other foods such as eggs and seafood. A high proportion (54%) of the vegetables used by our sample was not considered “carotenoid-rich”. These included common vegetables in the American diet such as potatoes, onions, celery, cauliflower, eggplant, and squash. Further, as some carotenoid-rich vegetables such as greens are often served raw as opposed to cooked, lower overall volume may limit carotenoid density. Our finding that total carotenoid content was not associated with total fruit and vegetable servings is in line with previous research. Pitts et al. noted a similar discrepancy among African Americans in North Carolina, such that while skin carotenoids were significantly associated with carotenoid intake and plasma carotenoids, they were not associated with total self-reported fruit and vegetable intake (excluding potatoes) [19]. These findings highlight that specific messages focused on increasing red, orange, and dark green vegetables may be necessary to increase the intake of carotenoid-rich produce.

Participants that prepared meals with higher amounts of carotenoids appeared to use more canned vegetable products, particularly tomato-based products. This finding is in line with research suggesting some of the main dietary sources of lycopene include pizza and pasta [26]. Given the ubiquity of processed tomato and other vegetable products in the American food system and the high carotenoid content of many such products, cooking education programs could refocus from avoiding canned or packaged foods to focusing on techniques and products that minimize added salt, sugar, and fat. Further, canned vegetables and tomato sauces may be more shelf-stable, cost-efficient, and require less preparation time than fresh products.

The use of processed foods is not without issue. As our data demonstrated, high carotenoid content meals were also high in sugar and refined grains. This is not surprising given that many pasta sauces and tomato products have sugar added to them to create product consistency, and sauces are typically served with refined grain pastas. Culinary education for health should consider highlighting appropriate versions of processed foods (i.e., low-sugar varieties) and optimal usage (whole grain or vegetable alternatives to refined grain pasta), especially in the context of cancer prevention and survivorship. Healthy cooking curricula may also include a focus on fresh carotenoid-rich foods as side dishes. One meal with both a high carotenoid and high HCI score included a kale and strawberry salad side dish, and another included a sweet potato side dish, both of which boosted total carotenoid content (Table 4).

Childhood cancer survivors may gain particular benefit from healthy cooking programs as they are both at increased risk for diet-related disease, and consistently do not meet recommended guidelines for fruit and vegetable intake [31,32]. Enhancing carotenoid intake for survivors may help mitigate late-effects of some cancer treatments such as cardiovascular disease and secondary cancers [33,34,35]. Although more research is needed to examine the specific role of carotenoids in pediatric cancer treatment and survivorship, enhancing fruit and vegetable intake in this population (processed or fresh) will enhance overall dietary patterns and support long-term survivor wellness.

This study has several strengths including the use of observed cooking behavior and nutritional data that is not subject to the reporting or recall bias of self-report approaches typically used in nutritional assessment [36,37]. This is also the first study to examine family cooking practices typically taught in modern cooking education programs relative to carotenoid content of prepared meals. Limitations of this study include the small, homogenous sample size. Information on typical diet was not collected and was limited only to the observation event. The HCI is currently only scored with a +1/−1 system, limiting our understanding of how nuances of these individual behaviors predict meal composition. Also, carotenoid bioavailability is influenced by several factors including food processing and meal composition [38], which are not captured by the HCI. The HCI was not constructed specifically to support high carotenoid usage or bioavailability during home cooking, and is therefore limited in its current form as a tool for scoring carotenoid content of meals. However, the current work could be built upon to develop a more carotenoid-specific healthy cooking framework. Carotenoids are not the only important components of survivor health and the impact of cooking practices on other micronutrients and phytochemicals should be continually explored. Future research should consider integrating biological measures of serum or dermal carotenoids in the assessment of community cooking and nutrition programs and integrating measures of phytochemicals and other micronutrients relevant to survivorship.

As cooking programs for children become more popular in K-12 schools, children’s camps, and pediatric hospitals, there is a clear opportunity to target programs for childhood cancer survivors and their families [10,11]. Cooking classes have the potential to translate nutrition recommendations for pediatric cancer survivors and their families to real-world food preparation scenarios, and existing teaching kitchen infrastructure may be used to serve this group. However, session curricula should be carefully structured and objectively examined in order to maximize the full spectrum of nutrients and phytochemicals potentially impacted by class teachings. While from-scratch cooking is an important skill, cooking programs may also consider the integration of low-sugar processed foods that deliver carotenoids through the diet and, in turn, support long-term survivor health.

## Figures and Tables

**Table 1:** Participant demographic and meal carotenoid characteristics.

Variable		% (*n*)
Child age (years)	5 to 8	42.5 (17)
	9 to 13	45.0 (18)
	14 to 18	12.5 (5)
Child sex	Male	35.0 (14)
	Female	65.0 (26)
Child race/ethnicity	Non-Hispanic white	40 (16)
	Hispanic white	27.5 (11)
	Non-Hispanic black	17.5 (7)
	Other	10.0 (4)
	Asian	5.0 (2)
Parent female		95.0 (38)
Parent age mean, SD (range)		39.9, 6.4 (28–56)
# Children in home mean, SD (range)		2.28, 0.99 (1–5)
Fruit/Veg servings per serving of prepared meal mean +/− SD (range)		2.74 +/− 2.07 (0–9.47)
Carotenoid content per serving of prepared meal mcg mean +/−SD (range)	Beta carotene	1867 +/− 2275 (0–11,233)
	Alpha carotene	255 +/− 346 (0–1345)
	Beta cryptoxanthin	50 +/− 75 (0–308)
	Lutein + zeaxanthin	1219 +/− 2270 (0–13,866)
	Lycopene	3416 +/− 267 (0–17,156)
	Total carotenoids	6807 +/− 7329 (9–33,624)

**Table 2:** Associations between individual carotenoids and the carotenoid-rich vegetable subgroup (Pearson correlation).

	Dark Green Vegetables	Tomato	Deep Yellow Vegetables
**Beta-carotene**	0.459 **	0.109	0.738 **
**Alpha-carotene**	0.324 *	−0.137	0.252
**Beta-cryptoxanthin**	0.053	0.083	−0.057
**Lutein + zeaxanthin**	0.473 **	0.128	−0.006
**Lycopene**	−0.163	0.865 **	−0.165

** Correlation is significant at the 0.01 level (2-tailed). * Correlation is significant at the 0.05 level (2-tailed).

**Table 3:** Associations between combined carotenoids and fruit and vegetables (Pearson correlation).

	Total F/V	Vegetables ^a^	Fruits	Total Carotenoid-Rich Vegetables
**Total Carotenoids**	0.14	0.16	−0.07	0.55 **

**. Correlation is significant at the 0.01 level (2-tailed). ^a^ includes potatoes.

**Table 4:** Examples of high and low carotenoid content (mcg/serving) meals across categories of prepared dishes, macronutrients (g) and observed Healthy Cooking Index Score (HCI).

Dish Category	Carotenoid Content, High/Low	Description	Total Carotenoids	Beta- Carotene	Alpha- Carotene	Beta-Cryptoxanthin	Lutein and Zeaxanthin	Lycopene	Calories	Total Fat	Protein	Carb	HCI
**Pasta**	High	Regular spaghetti with ground beef, jarred spaghetti sauce (with cheese) and green peppers + side of canned spinach and frozen garlic bread	33,624	8111	13	1	13,866	11,633	949	44.4	53.1	85.5	−1
	Low	Protein-enhanced spaghetti with garlic, fresh shrimp and olive oil + side of roasted broccoli	1182	412	13	1	756	0	833	39.8	48.0	81.5	6
**Stir Fry**	High	Multi-vegetable stir fry including pork sausage, fresh orange/yellow bell pepper and garlic, flavored with ginger and soy sauce + side of white rice	7289	3693	1345	1	2250	0	595	22.0	17.5	83.1	1
	Low	Multi-vegetable stir fry with skinless chicken breast, frozen broccoli-carrot-cauliflower-pepper blend, and fresh green beans flavored with stir fry sauce + side of brown rice	1183	547	148	9	479	0	304	12.4	16.0	32.9	5
**Tacos**	High	“Paleo” [30] tacos with ground beef, canned tomatoes, black beans and guacamole (premade) on cassava tortillas	18,434	1177	174	34	490	16,559	734	31.5	44.2	72.3	−1
	Low	Fish tacos with swai fish, lettuce, cilantro, jarred salsa and sour cream on plain flour tortillas	355	166	3	20	166	0	694	48.9	25.6	38.4	0
**Chicken**	High	Chicken breast sautéed with fresh cherry tomatoes + side of kale/strawberry salad and side of green pasta with butter and cheese	3228	1377	63	16	283	1489	472	20.3	31.4	42.2	6
	Low	Deep-fried skinless chicken breast + side of boudin sausage	9	0	0	0	9	0	936	54.1	69.6	38.2	−4
**Fish**	High	Roasted salmon with salt and pepper + side of microwaved sweet potatoes and steamed broccoli	11,918	11,233	21	0	664	0	309	11.3	22.6	29.3	4
	Low	Roasted catfish with bread crumbs and aioli sauce + side of frozen broccoli–carrot–squash blend, chicken bouillon and butter	1508	808	264	14	440	0	613	37.4	29.9	39.7	4
**Stew/Chili**	High	Packaged chili mix made from spice packets, ground beef, diced tomatoes, tomato sauce and canned beans + side of white rice	12,597	1580	183	308	45	10,481	744	25.1	39.5	89.9	−2
	Low	Shrimp stew made from sinigang spice packet mix, fresh eggplant, fresh tomato, long beans, ginger and taro root + side of white rice	2760	794	101	10	661	1194	479	1.7	47.1	71.2	6

**Table 5:** Associations between nutrient variables and total carotenoid content, carotenoid-rich fruit/vegetables, and total fruit/vegetables (Pearson correlation).

Nutrition Variable	Total Carotenoids	Total Carotenoid Rich Fruit/Vegetables	Total Fruit/Vegetables ^a^
Energy density (calories/grams)	−0.235	−0.278	−0.592 **
Sugar (g)	0.324 *	0.127	0.462 **
Fiber (g)	0.149	0.032	0.580 **
Meal servings of refined grains	0.497 **	0.199	−0.081
Meal servings of whole grains	−0.293	−0.315 *	−0.164
Meal servings of sweets	0.449 **	0.087	0.163
Sodium (mg)	0.099	0.116	0.348 *
Vegetable protein (g)	0.483 **	0.171	0.343 *
Vitamin A (mcg)^b^	0.539 **	0.616 **	0.215
Vitamin K (mcg)	0.509 **	0.445 **	0.347 *
Vitamin C (mcg)	−0.026	0.330 *	0.704 **
Vitamin B6 (mcg)	0.222	0.166	0.328 *
Folate (mcg)	0.723 **	0.388 *	0.312
Vitamin B12 (mcg)	0.189	0.034	0.170
Calcium (mg)	0.041	−0.120	0.450 **
Magnesium (mg)	0.179	0.174	0.560 **
Iron (mg)	0.658 **	0.258	0.388 *
Copper (mg)	0.303	0.408 **	0.577 **
Selenium (mcg)	0.373 *	−0.020	0.082
Potassium (mg)	0.293	0.168	0.783 **
Choline (mg)	−0.108	−0.199	0.347 *

** Correlation is significant at the 0.01 level (2-tailed). * Correlation is significant at the 0.05 level (2-tailed). ^a^ Includes potatoes. ^b^ Total Retinol Activity Equivalents.
